# Magnetic Nanomaterials for Hyperthermia-Based Therapy, Imaging, and Drug Delivery

**DOI:** 10.3390/pharmaceutics16020263

**Published:** 2024-02-11

**Authors:** Constantin Mihai Lucaciu

**Affiliations:** Department of Pharmaceutical Physics-Biophysics, Faculty of Pharmacy, “Iuliu Hatieganu” University of Medicine and Pharmacy, 6 Pasteur St., 400349 Cluj-Napoca, Romania; clucaciu@umfcluj.ro; Tel.: +40-744647854

In recent years, nanomedicine has experienced remarkable advancements, due to the development of new nanomaterials with outstanding properties that have demonstrated significant advantages over traditional medicines.

Among various nanoparticles, magnetic nanoparticles (MNPs) have emerged as promising candidates for a wide range of biomedical applications, particularly due to their responsiveness to external magnetic fields, enabling remote control and manipulation [1]. Their first medical applications were related to their role in changing water proton magnetic relaxation times, being used as contrast agents in magnetic resonance imaging (MRI) [2]; iron oxide MNPs are FDA-approved for imaging purposes and anemia treatments. While numerous studies are dedicated to improving the contrast in both T_1_ and T_2_ weighted MRI images using MNPs, magnetic particle imaging (MPI) has emerged in the last decade as an alternate technique, with tremendous potential in medical imaging [3], featuring multiple advantages such as linear quantitation, no tissue background, deep penetration, and no use of ionizing radiation.

Another important medical application of MNPs in the treatment of solid cancer tumors is magnetic hyperthermia (MH), a technique based on the heating of MNPs when submitted to radiofrequency alternating magnetic fields upon their loading in the tumor area. MH has achieved significant recognition, representing the first ever medical device to receive European approval for the treatment of glioblastoma multiforme (GBM), a form of brain tumor. Furthermore, ongoing clinical trials for U.S. Food and Drug Administration (FDA) approval are under way, specifically focusing on the treatment of prostate and pancreatic cancers. Despite the promise demonstrated in clinical trials where MH therapy was combined with standard chemotherapy and/or radiotherapy, achieving complete tumor regression remains elusive. Various factors contribute to this outcome, with the heat performance of injected MNPs playing a pivotal role. Therefore, a large number of studies have been conducted in the past few decades aiming to understand the role of composition, shape, size, structure, and surface functionalization in the heating performance of MNPs in MH [4]. Direct heat effects contribute to cell death; however, the elevated temperatures of MNPs open avenues for further exploration. In many preclinical and clinical studies, authors have initially hypothesized that the therapeutic impact observed was primarily attributed to the induction of tumor necrosis during hyperthermia applications. However, the potential effects on the tumor microenvironment, specifically the induction of anti-tumor immune responses, have not been thoroughly examined. Contemporary perspectives now suggest that a crucial element contributing to the therapeutic efficacy of hyperthermia is the initiation of a heat-mediated immune response against the tumor. This immune response enhances the tumor’s visibility to the immune system, suggesting that MH treatment can facilitate spatially and temporally controlled temperature increases, acting as an immune trigger. This recognition of hyperthermia as an immune modulator underscores its potential in not only targeting tumors directly, but also in harnessing the body’s immune system for a more comprehensive and effective therapeutic approach [5]. This highlights the evolving landscape of MNPs as a promising tool in the realm of cancer therapeutics.

By surface functionalization or through the creation of hybrid formulations with polymers, fluorophores, liposomes, and plasmonic or silica shells, MNPs acquire multifunctional capabilities. These multifaceted nanomaterials hold the potential to both diagnose and treat medical conditions, offering the ability to remotely control their positioning and activation, thus positioning them as a highly investigated class of theranostic materials. Among the numerous applications of such hybrid nanostructures, here we focus on controlled drug delivery. Drug delivery systems utilizing MNPs present a range of advantages, enhancing the precision and efficiency of therapeutic interventions. Some key benefits include the ability to target specific anatomical locations within the body, a reduction in the required drug dosage to achieve a specific concentration at the target site, and the mitigation of potential side effects. By leveraging the magnetic properties of MNPs, these delivery systems enable a more focused and controlled release of therapeutic agents, optimizing the therapeutic outcome while minimizing the impact on non-target sites. This targeted drug delivery approach holds promise for enhancing treatment efficacy and minimizing undesirable effects associated with systemic drug administration.

This Special Issue, entitled “Magnetic Nanomaterials for Hyperthermia-Based Therapy, Imaging, and Drug Delivery”, provides a platform for researchers to share their latest experimental and theoretical findings related to the development and application of magnetic nanomaterials in the medical field, assembling both reviews (four) and research papers (seven) focused on the distinct aspects of MNP medical applications and written by recognized experts in the field.

In the first review paper, Gago et al. (contribution 11) conducted a systematic search of in vitro and in vivo studies published in the last decade that employ hyperthermia therapy mediated by magnetic nanoparticles for treating gastrointestinal cancers. The results revealed that iron oxide is the preferred material for magnetism generation in nanoparticles, and colorectal cancer is the most widely studied gastrointestinal cancer. Interestingly, novel therapies employing nanoparticles loaded with chemotherapeutic drugs, in combination with magnetic hyperthermia, demonstrated an excellent antitumor effect.

Ganguly and Margel (contribution 9) explored the most recent developments in synthetic methodologies and methods for the fabrication of magneto-fluorescent nanocomposites. The primary applications of multimodal magneto-fluorescent nanoparticles in biomedicine, including biological imaging, cancer treatment, and drug administration, are covered in this article, together with an overview of the future possibilities for these technologies.

Pusta et al. (contribution 8) performed a critical overview of the recent literature concerning advancements in the field of magnetic nanoparticles used in drug delivery, with a focus on their classification, characteristics, synthesis and functionalization methods, limitations, and examples of magnetic drug delivery systems incorporating chemotherapeutics or RNA.

Chan et al. (contribution 10) reviewed potential improvements in the magnetic properties of iron-based nanoparticles in the preparation of multifunctional composite materials through their combination with ceramic materials. They demonstrate the potential of ferromagnetic enhancement and multifunctional composite materials for MRI diagnosis, drug delivery, MH therapy, and cellular imaging applications.

An interesting study was reported by Alsenousy et al. (contribution 7) on the potential of superparamagnetic iron oxide nanoparticles (SPIONs) as anti-obesity agents. For the first time, the authors reported promising ameliorating effects of SPION treatments against weight gain, hyperglycemia, adiponectin, leptin, and dyslipidemia in obese rats. At the molecular level, surprisingly, SPION treatments markedly corrected the disturbed expression and protein content of inflammatory markers and parameters controlling mitochondrial biogenesis and functions in both brown and white adipose tissue.

The combination of magnetic hyperthermia with chemotherapy is considered a promising strategy in cancer therapy due to the synergy between high temperatures and chemotherapeutic effects, which can be further developed for targeted and remote-controlled drug release. Nitica et al. (contribution 6) reported a simple, rapid, and reproducible method for the preparation of thermosensitive magnetoliposomes (TsMLs) loaded with doxorubicin (DOX), consisting of a lipidic gel formation from a previously obtained water-in-oil microemulsion with fine aqueous droplets containing magnetic nanoparticles (MNPs) dispersed in an organic solution of thermosensitive lipids (transition temperature of ~43 °C), followed by gel hydration with an aqueous solution of DOX. The obtained thermosensitive magnetoliposomes (TsMLs) were around 300 nm in diameter and exhibited 40% DOX incorporation efficiency. The most suitable MNPs to incorporate into the liposomal aqueous lumen were Zn ferrites, with a very low coercive field at 300 K (7 kA/m), close to the superparamagnetic regime, exhibiting a maximum specific absorption rate (SAR) of 1130 W/gFe when dispersed in water and 635 W/gFe when confined inside TsMLs. No toxicity of Zn ferrite MNPs or TsMLs was noticed against the A459 cancer cell line after 48 h incubation over the tested concentration range. The passive release of DOX from the TsMLs after 48 h incubation induced toxicity, starting with a dosage level of 62.5 mg/cm^2^. Below this threshold, the subsequent exposure to an alternating magnetic field (20–30 kA/m, 355 kHz) for 30 min drastically reduced the viability of the A459 cells due to the release of incorporated DOX. These results strongly suggest that TsMLs represent a viable strategy for anticancer therapies using the magnetic-field-controlled release of DOX.

Oliveira et al. (contribution 5) reported the development of paclitaxel-loaded lipid-coated manganese ferrite magnetic nanoparticles (PTX-LMNPs) as synthetic magnetosome analogs, envisaging the combined chemo-magnetic hyperthermia treatment of melanoma. Their results showed that PTX-LMNP-mediated MHT triggers PTX release, facilitating its thermal-modulated local delivery to diseased sites within short timeframes. Moreover, half-maximal PTX inhibitory concentration (IC_50_) could be significantly reduced relatively to free PTX (142,500×) and Taxol^®^ (340×). Therefore, the dual chemo-MHT therapy mediated by intratumorally injected PTX-LMNP stands out as a promising alternative to efficiently deliver PTX to melanoma cells, consequently reducing systemic side effects commonly associated with conventional chemotherapies.

Unak et al. (contribution 4) obtained MNPs surrounded by silica and an organic layer, labeled with ^44^Sc for SPECT and ^47^Sc for radiotherapy. The radiobioconjugate exhibited high affinity and cytotoxicity toward the human prostate cancer LNCaP (PSMA+) cell line, much higher than for PC-3 (PSMA-) cells. High cytotoxicity of the radiobioconjugate was confirmed by radiotoxicity studies on LNCaP 3D spheroids. In addition, the magnetic properties of the radiobioconjugate should allow for its use in guiding drug delivery driven by a magnetic field gradient.

Caizer et al. (contribution 3) reported an in vitro study on the human breast adenocarcinoma cell line (MCF-7) by applying superparamagnetic hyperthermia (SPMHT), using novel Fe_3_O_4_-PAA–(HP-γ-CDs) (PAA is polyacrylic acid and HP-γ-CDs is hydroxypropyl gamma-cyclodextrins) nanobioconjugates, obtaining >95% cell deaths in specific alternating magnetic field conditions.

Chircov et al. (contribution 2) presented a microfluidic device to obtain a series of antibiotic-loaded MNPs. The results proved a considerable uniformity of antibiotic-containing nanoparticles, good biocompatibility, and promising antimicrobial properties against *S. aureus*, *P. aeruginosa*, and *C. albicans* strains.

Bernad et al. (contribution 1 investigated the agglomeration processes of magnetoresponsive functionalized nanocluster suspensions in a magnetic field, as well as how these structures impact the behavior of these suspensions in biomedical applications. Their results show that the applied magnetic field aligns the magnetic moments of the nanoclusters, resulting in the formation of chains, linear aggregates, or agglomerates of clusters aligned along the applied field direction. The design of chain-like structures can cause considerable changes in the characteristics of ferrofluids, ranging from rheological differences to colloidal stability changes.

The diverse topics covered in this Special Issue span the spectrum from drug delivery to hyperthermia-based therapy and cancer and other disease treatments, showcasing the versatility of magnetic nanoparticles in addressing multifaceted challenges in medicine.

A notable aspect highlighted in the contributions is the potential for synergistic effects, particularly in conjunction with other therapeutic modalities such as chemotherapy. This emphasizes the pivotal role that magnetic nanomaterials can play in shaping the landscape of medical treatments.
